# Epithelial Membrane Protein 1 Promotes Sensitivity to RSL3-Induced Ferroptosis and Intensifies Gefitinib Resistance in Head and Neck Cancer

**DOI:** 10.1155/2022/4750671

**Published:** 2022-04-06

**Authors:** Ying Wang, Liang Zhang, Changyu Yao, Yunxia Ma, Yehai Liu

**Affiliations:** ^1^Department of Otorhinolaryngology, Head & Neck Surgery, The First Affiliated Hospital of Anhui Medical University, Hefei, China; ^2^Chair of Livestock Biotechnology, School of Life Sciences Weihenstephan, Technical University of Munich, Liesel Beckman Str 1, 85354 Freising, Germany

## Abstract

Epithelial membrane protein (EMP1), a member of the peripheral myelin protein (PMP22) family, is involved in the development of various human malignancies. However, the expression level of EMP1 and its functional role in head and neck squamous cell carcinoma (HNSCC) remain unclear to date. Ferroptosis, a newly characterized form of regulated cell death, plays an essential role in tumorigenesis. In this study, we aimed to investigate the expression levels of EMP1 in HNSCC and normal tissues, as well as to identify the function of EMP1 in regulating ferroptosis during the progression of HNSCC. To further explore the biological function of EMP1 in vitro, transient transfection was used to overexpress EMP1 in the HNSCC cell lines Hep2 and Detroit562. Functionally, our results indicated that EMP1 overexpression could not affect the initiation of ferroptosis directly but reinforced RSL3-induced ferroptosis on HNSCC cells. Furthermore, mechanical study indicated that EMP1 mediated the ferroptosis via cell density-regulated Hippo-TAZ pathway and regulated the expression of Rac1 and NOX1. In addition, our study demonstrated that EMP1 overexpression could promote gefitinib resistance by targeting the MAPK pathway. In summary, our findings indicate that EMP1 may act as an oncogene and serve as a therapeutic target against malignant progression of HNSCC.

## 1. Introduction

Globally, head and neck squamous cell carcinoma (HNSCC) ranks sixth in terms of morbidity among the most common types of malignancies with over 600,000 new cases annually [1]. HNSCC has a higher relative lethal rate due to its early asymptomaticity. Despite considerable advancement in the diagnosis and therapeutic schedule in the past few years, the average five-year survival rate is still less than 50% [2]. Therefore, it is important to investigate the pathogenesis of HNSCC and to identify new biomarkers for predicting prognosis that would help in the future to develop new therapeutic approaches.

Epithelial membrane protein 1 (EMP1) is encoded by the peripheral myelin protein (PMP22) family and is also known as a tumor-associated membrane protein. As the term suggests, EMP1 is primarily expressed in squamous epithelium and presents different expression levels in a variety of normal tissues, such as the esophagus, stomach, adipose, and gallbladder [3, 4]. In addition, a previous study showed that EMP1 in a variety of tumors, such as glioblastoma multiforme (GBM) [5], uveal melanoma [6], non-small-cell lung cancer (NSCLC) [7], and acute lymphoblastic leukemia (ALL) [8], exhibited increased expression, whereas EMP1 in nasopharyngeal cancer [9], gastrointestinal cancers [10], colorectal cancer [11], and ovarian cancer [12] show decreased expression. Besides, EMP1 acts as a key regulator of cell proliferation, migration, and death. The reduced expression of EMP1 has been reported to be associated with poor prognosis in patients with certain types of malignant tumors and regarded as an independent predictor of poor survival in various solid tumors [13, 14]. Additionally, it has been shown that overexpression of EMP1 leads to a reduction in the proliferation of nasopharyngeal tumor [10]. In breast cancer, overexpressed EMP1 inhibits migration and invasion by reducing the expression of vascular endothelial growth factor- (VEGF-) C [14]. However, the actual function of EMP1 is controversial. In most cancers, the tumor suppressive functions of EMP1 have been described as inhibition of cell growth and metastasis by induction of apoptosis and prevention of angiogenesis [9].

Collectively, these findings suggest that EMP1 may act as a promising therapeutic target in numerous types of human malignancies. To the best of our knowledge, there have so far been no reports on the expression and functional role of EMP1 in HNSCC.

Ferroptosis, as a newly identified form of programmed cell death, is an iron-dependent, oxidative cell death characterized by iron-dependent accumulation of reactive oxygen species (ROS) and decreased intracellular glutathione (GSH) levels. Lipid ROS can be accumulated by either the expression of glutathione peroxidase 4 (GPX4) or the generation of nicotinamide adenine dinucleotide phosphate (NADPH) oxidases (NOXs). The lipid peroxidase pathway is an essential metabolic pattern of ferroptosis in cancer cells [15, 16]. Yang and Stockwell discovered that some small molecules can activate iron-dependent cell death in cancer cells, such as RAS-selective lethal 3 (RSL3) and erastin [17]. Ferroptosis is morphologically, biochemically, and genetically distinct from other cell deaths, suggesting its therapeutic potential for HNSCC. Recently, sequence-based studies have shown that ferroptosis-related gene signature plays an important role in the development and treatment of HNSCC [18–20]. CAV1, which could inhibit ferroptosis, leads to cancer progression in HSNCC [21]. Li et al. reveal that IL-6 induces ferroptosis resistance by protecting tumor cells from ROS and promoting HNSCC progression [22]. Recent studies showed that targeting ferroptosis-associated metabolic pathway could improve the efficacy of cancer immunotherapy [23].

The present study is aimed at clarifying the potential impact of EMP1 on ferroptosis in HNSCC, and we found that EMP1 overexpression could reinforce RSL3-induced ferroptosis. In addition, we assessed the underlying signaling pathways of EMP1-mediated ferroptosis and examined the effects of overexpressed EMP1 on the gefitinib resistance in the HSCC cell line.

## 2. Material and Methods

### 2.1. Cell Culture and Transfection

The human HNSCC cell lines (Hep2, Detroit562, and TU686) and human nonmalignant nasopharyngeal epithelial cells NP69-SV40T were obtained from ATCC and cultured according to the standard ATCC protocol. In brief, cells were cultured in Dulbecco's modified Eagle's medium (DMEM) (Gibco, Carlsbad, CA, USA) containing 10% fetal bovine serum (FBS; HyClone, Logan, UT, USA). The cells were maintained at 37°C in a humidified atmosphere of 5% CO_2_ and were used during their logarithmic growth phase. Full-length cDNAs of human EMP1 was obtained by reverse transcription PCR using RNA extracted from human cultured cells. And then EMP1 cDNA sequence was cut out and inserted in the pcDNA3.1-vector (Thermo Fisher Scientific, Waltham, MA, USA). The Hep2 and Detroit562 cells were stably transfected with pcDNA3.1-EMP vector.

### 2.2. Cell Treatment

The Hep2 and Detroit562 cell lines were stimulated with the indicated doses of ferroptosis agonist erastin, RSL3. Furthermore, the cells were treated with Rac1 activator phorbol 12-myristate 13-acetate (PMA) and Rac1 inhibitor NSC 23766, respectively. The cells were cultured with stepwise escalation of concentration of gefitinib from 2.5 nM to 50 *μ*M.

### 2.3. RNA Isolation and Quantitative Real-Time PCR

Total RNA was extracted from Hep2 and Detroit562 cells using TRIzol reagent (Invitrogen) according to the manufacturer's protocol. Quantitative rea-time PCR (qRT-PCR) was conducted with an ABI Prizm 7300 (Applied Biosystems Inc., Carlsbad, CA, United States) according to the standard protocol for SYBR Premix ExTaq (Perfect Real Time; TakaRa). The following are the primers used: EMP1—5′-CATGCTGTTCGTTTGCACCA-3′ (forward), 5′-TTCACCGCCGTATAACAGGG-3′ (reverse); GAPDH—5′-AACGGATTTGGTCGTATTGG-3′ (forward), 5′-TTGATTTTGGAGGGATCTCG-3′ (reverse); TAZ—5′-TGC TAC AGT GTC CCC ACA AC-3′ (forward), 5′-GAA ACG GGT CTG TTG GGG AT-3′ (reverse); YAP1—5′-CAACTCCAACCAGCAGCAAC-3′ (forward), 5′-TTGGTAACTGGCTACGCAGG-3′ (reverse); Rac1—5′-TGCCGATGTGTTCTTAATTTGC-3′ (forward), 5′-CTTCTTCTCCTTCAGTTTCTCGATC-3′ (reverse); NOX1—5′-ATGGGAAACTGGGTGGTTAAC-3′ (forward), 5′-CCTATAACTCAAAAATTTTCTT-3′ (reverse).

### 2.4. Cell Viability

Cell viability and cell death after exposure to EMP1 were measured by the CCK-8 assay. In brief, HNSCC cells in the logarithmic growth period were collected and dispensed into 96-well culture plates (5 × 10^3^ cells/well) following the different interventions. 10 *μ*L CCK-8 solution was added to the culture plates and incubated continuously for 4 h. The absorbance was measured utilizing a microplate reader (Varioskan Flash, Thermo Scientific) spectrophotometer at the wavelength of 490 or 450 nm.

### 2.5. Immunofluorescence

ROS generation level in the HNSCC cell line lysates was assessed by oxygen-free radical and lipid peroxidation. Immunofluorescence was performed as the recommended protocol. Briefly, HNSCC cells were subjected to test compounds for the indicated times; after being washed twice in PBS, cells were fixed on coverslips by methanol for 5 min at RT. After fixation, cells were washed three times in PBS for 5 min and treated with a blocking solution (1% BSA in PBS) for 30 min. Subsequently, the cells were washed twice in PBS and incubated with the primary antibody for 30 min. The level of oxygen-free radical and lipid peroxidation was determined using 2′,7′-dichlorodihydrofluorescein diacetate (DCFH-DA, 20 *μ*M, Sigma) and BODIPY™ 581/591 C11 (2 *μ*M, D3861; Thermo Fisher), respectively. Nuclei were stained with DAPI Stain Solution. Confocal microscopy was used to detect the fluorescence, and the fluorescence intensity of DCF was tested by SpectraMax iD3.

### 2.6. Flow Cytometer

The level of oxygen-free radical and Lipid Peroxidation was determined using 2′,7′-dichlorodihydrofluorescein diacetate (DCFH-DA, Sigma) and BODIPY™ 581/591 C11 (D3861; Thermo Fisher), respectively. Oxidation of the polyunsaturated butadienyl portion of C11 BODIPY resulted in a shift of the fluorescence emission peak from ~590 nm to ~510 nm proportional to lipid peroxidation generation. After next incubation for 30 minutes at 37°C, a flow cytometer was then used to analyze the signal of these samples and data were collected from the FL1 channel; software analysis was carried out using FlowJo v10.

### 2.7. Western Blot Analysis

To evaluate related protein level after treatment, HNSCC cells were collected and lysed by RIPA lysis buffer. Proteins with equal concentration were subjected to the SDS-PAGE and transferred to PVDF membrane (BIO RAD, USA). All samples were blocked by nonfat milk (5%) and incubated with primary antibodies overnight at 4°C. The primary antibodies included anti-EMP1 (Santa Cruz Biotechnology, CA, USA), anti-YAP (Cat, #ab52771), anti-TAZ (Cat, #560235), anti-nicotinamide-adenine dinucleotide phosphate (NADPH) oxidase 1 (NOX4) (Cat, #ab133303), anti-acyl-CoA synthetase 4 (ACSL4) (Cat, #ab155282), anti-GPX4 antibody (Cat. #ab125066), anti-Rac1 (Cat. #ab33186), anti-GAPDH (CST, #5176), and anti-nicotinamide-adenine dinucleotide phosphate (NADPH) oxidase 1 (NOX1) (Cat, #ab55831). Antibodies for phospho-epidermal growth factor receptor (EGFR) and EGFR were from Santa Cruz Biotechnology (Santa Cruz, CA). Phospho-AK, AKT, Phospho-ERK1/2, and ERK1/2 were from Cell Signaling Technology (Beverly, MA). On the next day, all membranes were incubated with the secondary antibody incubation for 2 hours at room temperature. The expression result was detected by ECL reagent (Sigma, USA, WBULS0100). Per condition analyzed a minimum of 10,000 cells.

### 2.8. Measurement of MDA Assay

Cellular lipid peroxidation in HNSCC cell line was evaluated by measuring the concentration of malondialdehyde (MDA). We used Lipid Peroxidation MDA Assay Kit (#ab118970, Abcam) based on thiobarbituric acid (TBA) reactivity. Briefly, after being treated as indicated, cells were harvested and lysed. Then, after protein quantification of the lysate, MDA working solution was added and heated at 100°C for 15 min. Next, the supernatant was collected after centrifuging at 1000 g for 10 min to remove debris and then measured at a 532 nm excitation wavelength. The relative cellular MDA concentration was expressed as a percentage of the absorbance value of the control.

### 2.9. Statistical Analysis

The data are presented as the mean ± standard deviation (SD). The statistical tests were performed using GraphPad prism 7.01 (GraphPad Software, Inc.). The Cancer Genome Atlas (TCGA) data of mRNA expression were downloaded from the University of California Santa Cruz (UCSC) Xena website (https://xenabrowser.net/datapages/). “Limma” package was then used to identify differentially expressed genes (DEGs). Next, DEGs were performed under the enrichment analysis of Gene Ontology (GO); among three parts (BP, CC, and MF) of this analysis, only MF (molecular function) was significantly enriched. Here, we set false discovery rate (FDR) < 0.05 and ∣log2(fold change) | >0.5 as the cut-off. Unless otherwise noted, *p* values were calculated using unpaired, two-tailed *t*-tests assuming unequal variance.

## 3. Results

### 3.1. Overexpression of EMP1-Induced Cell Death in HNSCC

**Figure 1:**
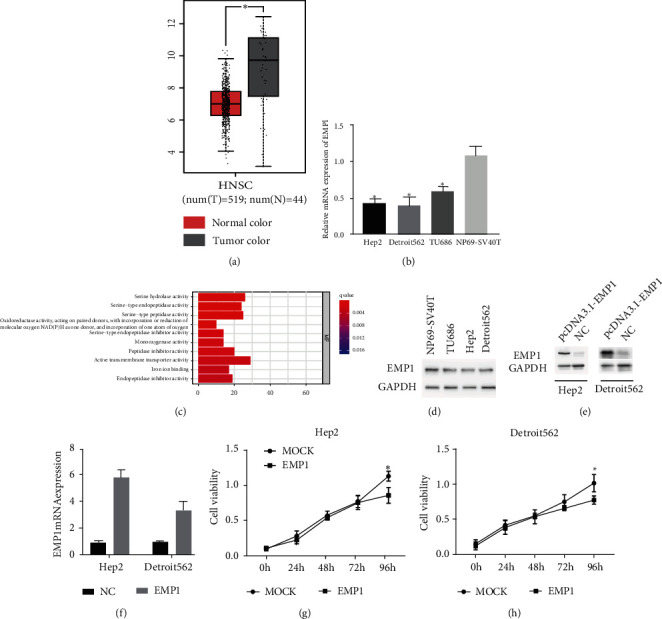
EMP1 inhibited cell growth in laryngeal cancer cells. (a) Comparison of EMP1 mRNA expression in HNSCC from TCGA databases. (b) EMP1 mRNA level in different HNSCC cell lines and human nonmalignant nasopharyngeal epithelial cells NP69-SV40T by qRT-PCR. (c) The list of significantly changed signaling pathways between comparison of EMP1-low and EMP1-high expression. The differentially expressed gens were collected based on the EMP1 expression by the median expression value of EMP1. (d) EMP1 protein in different HNSCC cell lines and human nonmalignant nasopharyngeal epithelial cells NP69-SV40T by Western blot. (e) Confirmation of EMP1 overexpression with protein level by in the human HNSCC cell lines Hep2 and Detroit562. (f) Confirmation of EMP1 overexpression with mRNA level by in the human HNSCC cell lines Hep2 and Detroit562. Effects of EMP1 on cell death. Cell viability was determined on days 0, 1, 2, 3, and 4 in the EMP1-overexpressing Hep2 (g) and Detroit562 cells (h). Relative viability normalized to the untreated condition was used. Values are the mean ± SD. ^∗^*p* < 0.05.

TCGA (https://portal.gdc.cancer.gov/) databases were utilized to investigate the expression of EMP1 in HNSCC, and significantly decreased EMP1 expression was observed in cancer tissues compared with normal tissues (*p* < 0.05) (Figure 1(a)). To validate this observation, EMP1 levels in different HNSCC cell lines and human nonmalignant nasopharyngeal epithelial cells NP69-SV40T were determined using qRT-PCR. As shown in Figure 1(b), the EMP1 level was considerably lower in HNSCC cell line when compared with those in NP69-SV40T, especially in Hep2 and Detroit562. To further explore the function of EMP1, the EMP1 expression levels were divided into EMP1-low (EMP1-L) and EMP1-high (EMP1-H) groups based on the median expression value of EMP1. The top ten significantly enriched GO pathways are listed in Figure 1(c). Among them, iron ion binding signaling pathways suggested a regulatory role of EMP1 in cell death. To assess the effect of EMP1 on cell death, we first selected suitable cell lines by testing EMP1 protein levels in NP69-SV40T and several HNSCC cell lines. Our results demonstrated that EMP1 expression was relatively low in Hep2 and Detroit562 cell lines (Figure 1(d)). Then, we used transient transfection with pcDNA3.1-EMP1 to overexpress EMP1. The overexpression efficiency was validated by WB and qRT-PCR, and the results indicated increased expression of EMP1 in the pcDNA3.1-EMP1 cells compared to control (Figures 1(e) and 1(f)). Treat the cells Hep2 and Detroit562; CCK-8 assays were conducted to detect the viability of HNSCC cells. After treatment, CCK-8 assays were performed to detect the viability of HNSCC cells. The results indicated that overexpressed EMP1 significantly inhibited cell growth in both EMP1-overexpressing cell lines in a time-dependent manner. The above results demonstrated that EMP1 inhibited cell growth in HNSCC (Figures 1(g) and 1(h)). EMP1 promoted RSL3-induced lipid peroxidation production and increased sensitivity to ferroptosis.

**Figure 2:**
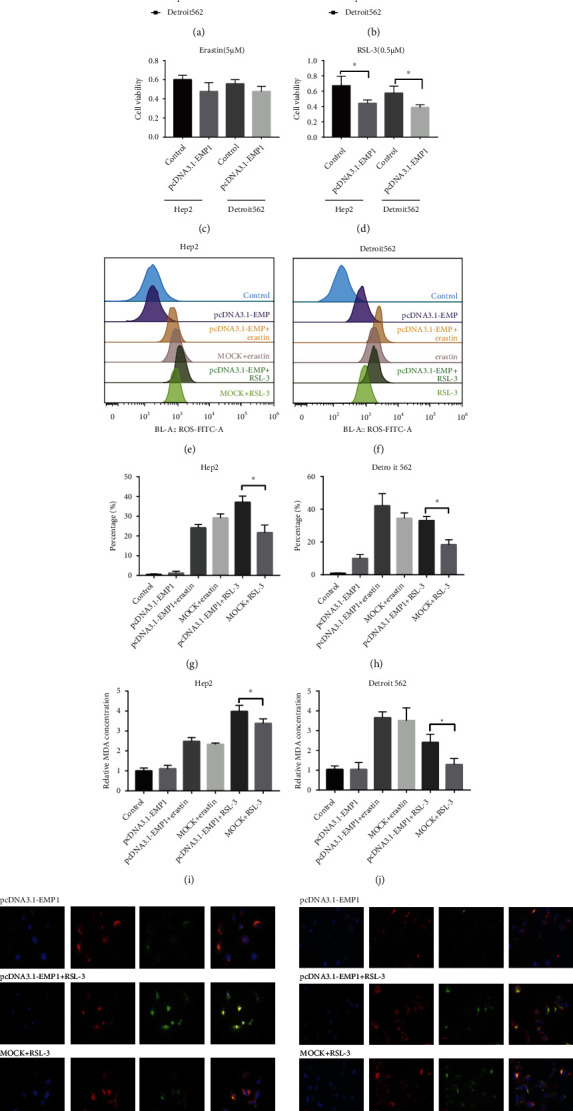
EMP1 promoted RSL3-induced lipid peroxidation production and increased sensitivity to ferroptosis EMP1 improved lipid oxidation. Cell viability in the MOCK-and EMP1-overexpressing Hep2 and Detroit562 cells after the treatment with different dose erastin (a) and RSL3 (b). Cell viability of Hep2 and Detroit562 cells after treatment with erastin (5 *μ*M) (c) and RSL3 (0.5 *μ*M) (d) was determined using CCK-8 assay kit. Lipid peroxidation was assessed with C11-BODIPY by flow cytometry in the MOCK- and EMP1-overexpressing Hep2 (e) and Detroit562 (f) cells pretreated with erastin (5 *μ*M) and RSL3 (0.5 *μ*M) or not. The percentage of expressing lipid peroxidation was assessed with C11-BODIPY by flow cytometry in the MOCK- and EMP1-overexpressing Hep2 (g) and Detroit562 (h) cells pretreated with erastin (5 *μ*M) and RSL3 (0.5 *μ*M) or not. MDA levels were detected using a lipid peroxidation MDA assay kit in the MOCK-and EMP1-overexpressing Hep2 (g) and Detroit562 (h) cells pretreated with erastin (5 *μ*M) and RSL3 (0.5 *μ*M) or not. (i, j) Fluorescence staining of cellular C11-BODIPY. Confocal microscopy showed the nonoxidized lipid (red) and oxidized lipid (green) in the MOCK- and EMP1-overexpressing Hep2 (k) and Detroit562 (l) cells pretreated with RSL3 (0.5 *μ*M) or not. Values are the mean ± SD. ^∗^*p* < 0.05.

To explore the role of EMP1 in regulating cellular oxidative status, we first detected cells' sensitivity to two widely used ferroptosis inducers by inhibiting the PL-peroxidase activity of GPX4, RSL3, and erastin. To determine the suitable concentration, we performed concentration gradient assays. Cell viability assay revealed that both erastin and RSL3 remarkably suppressed cell viability of HNSCC in a dose-dependent manner (Figures 2(a) and 2(b)). Erastin/RSL3 (5 *μ*M/0.5 *μ*M) were then used to treat cells, and their inhibition effects were determined by CCK-8 assay. Indeed, overexpression of EMP1 could robustly sensitize Hep2 and Detroit562 cell to RSL3-induced ferroptosis, but not erastin-induced ferroptosis (Figures 2(c) and 2(d)). Increased cellular ROS has been reported to cause lipid peroxidation. As indicated, FACS analysis following lipid peroxidation probe BODIPY™ 581/591 C11 staining in Hep2 cell line showed that increase was detected in the EMP1-overexpressing cells after being treated with RSL3 (Figures 2(e) and 2(g)) and Detroit562 (Figures 2(f) and 2(h)). To further demonstrate lipid peroxidation, MDA, which is a metabolic product of lipid peroxidation, was evaluated in the cells. The increased MDA production strongly indicated increased lipid peroxidation in the EMP1-overexpression cells lines after the treatment of RSL3 (Figures 2(i) and 2(j)). Moreover, an increased level of ROS was detected by immunofluorescence assays in EMP1-overexpressing cells when treatment with of RSL3 (Figures 2(k) and 2(l)). These results strongly indicated that EMP1 was involved in ferroptosis by promoting RSL3-induced lipid peroxidation production and increased sensitivity to ferroptosis.

### 3.2. Cell Density-Dependent Death Regulated EMP1 Sensitivity to Ferroptosis

**Figure 3:**
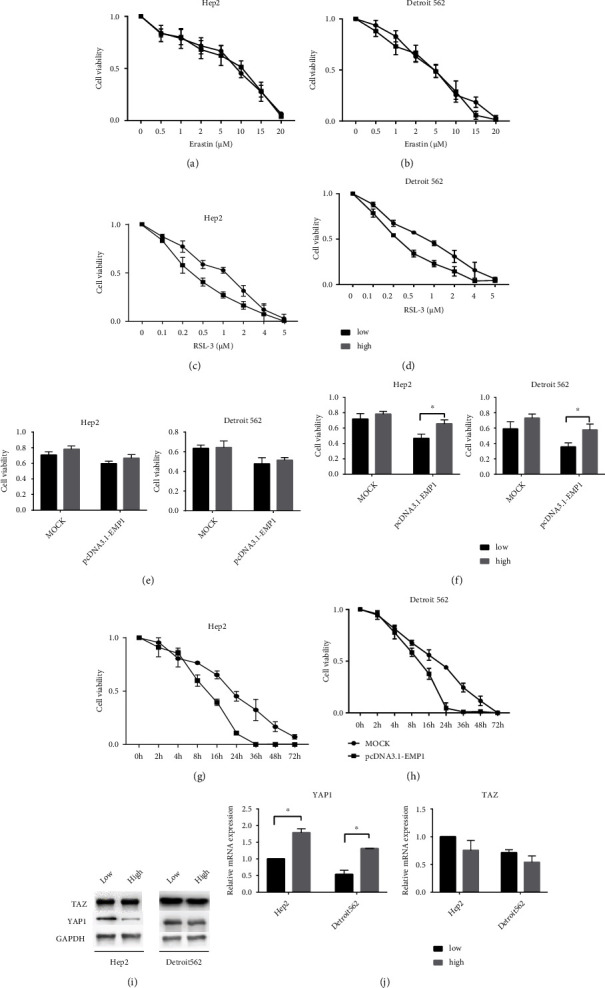
Cell density-dependent death regulated EMP1 sensitivity to ferroptosis. Cell viability in Hep2 (a) and Detroit562 (b) cells after the treatment with different dose erastin seeded in low/high densities. Cell viability in Hep2 (c) and Detroit562 (d) cells after the treatment with different dose RSL3 seeded in low/high densities. Cell viability in the MOCK-and EMP1-overexpressing Hep2 and Detroit562 cells after the treatment with erastin (5 *μ*M) (e) and RSL3 (0.5 *μ*M) (f) seeded in low/high densities. Cell viability in the MOCK-and EMP1-overexpressing Hep2 (g) and Detroit562 (h) cells after the treatment with RSL3 (0.5 *μ*M) at the indicated time. (i) YAP1 and TAZ level in Hep2 and Detroit562 cells check by western blot. (j) YAP1 and TAZ level in Hep2 and Detroit562 cells check by qRT-PCR at low/high densities. Values are the mean ± SD. ^∗^*p* < 0.05.

EMP1 mRNA was reported downregulated upon TAZ knockdown, and thus, TAZ activation may contribute to the density-dependent ferroptosis sensitivity [24]. To investigate whether overexpressed EMP1 regulates the RSL3-induced ferroptosis sensitivities under different cell densities, we first investigate the sensitivity of erastin and RSL3 at low and high densities. The cell viability showed the same sensitivity to erastin between the low and high groups (Figures 3(a) and 3(b)). In contrast, RSL3 sensitivity was negatively correlated with cell density. At low density, cells showed higher sensitivity to RSL3 compared with high density (Figures 3(c) and 3(d)). Next, we questioned if the increased sensitivity to ferroptosis at low density was related to EMP1 expression, and to answer this question, we treated these cells with erastin (5 *μΜ*) and RSL3 (0.5 *μΜ*) separately. As expected, cell viability results showed that erastin-induced cell death activities were the same among all cell densities in both the MOCK- and EMP1-overexpressing cell lines (Figure 3(e)). However, there was more RSL3-induced cell death at low density comparing high density in the overexpression EMP1 cell line (Figure 3(f)). To validate the efficacy duration of RSL3, we performed time-course experiment. The cell death curve showed that the speed of cell death displayed a time-dependent manner, indicating that the decreased cell density enhanced the rate of death (Figures 3(g) and 3(h)). These data indicated that EMP1 was a key factor responsible for cell density-dependent RSL3-induced cell death. Furthermore, EMP1 overexpression reduced the ferroptosis protection conferred by TAZ knockdown, indicating that EMP1 genetically works downstream of TAZ to regulate ferroptosis. Besides, YAP1 was also an upstream target responsible for RSL3-induced cell death in the EMP1-overexpressing cells. To further explore the main protein regulating ferroptosis via the cell density, we found that a comparable upregulation of YAP1 under high density while TAZ exhibited the same level under both low and high densities (Figure 3(i)). Further, qRT-PCR demonstrated that cell density did not affect TAZ mRNA levels in both Hep2 and Detroit562 cell lines indicating that YAP1 was a key factor responsible for RSL3-induced cell death in the EMP1-overexpressing cells (Figure 3(j)).

### 3.3. Rac1 Is a Direct Target Gene of ROS That Regulates Ferroptosis Sensitivity

**Figure 4:**
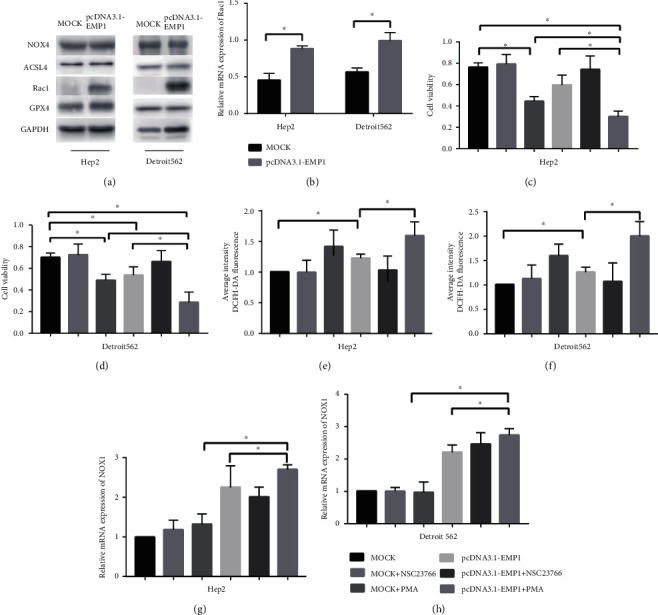
Rac1 is a direct target gene of ROS that regulates ferroptosis sensitivity. (a) Western blotting analyzed NOX4, ACSL4, Rac1, and GPX4 expression in the MOCK- and EMP1-overexpressing Hep2 and Detroit562 cells. (b) qRT-PCR analyzed the change of Rac1 mRNA in in the MOCK- and EMP1-overexpressing Hep2 and Detroit562 cells. Cell viability analysis in the MOCK- and EMP1-overexpressing Hep2 (c) and Detroit562 (d) cells pretreated with Rac1 activator PMA and Rac1 inhibitor NSC 23766, respectively. The intracellular ROS activity level was detected by average intensity DCFH-DA in the MOCK- and EMP1-overexpressing Hep2 (e) and Detroit562 (f) cells pretreated with Rac1 activator PMA and Rac1 inhibitor NSC 23766, respectively. qRT-PCR analyzed the change of NOX1 mRNA in the MOCK- and EMP1-overexpressing Hep2 (g) and Detroit562 (h) cells pretreated with Rac1 activator PMA and Rac1 inhibitor NSC 23766, respectively. Values are the mean ± SD. ^∗^*p* < 0.05.

To investigate the mechanistic link between EMP1 and ferroptosis, we sought to determine whether EMP1 would affect the levels of GPX4 or NOX4, two key regulators of lipid peroxidation in ferroptosis [25, 26]. Moreover, ACSL4 could regulate the sensitivity to RSL3-induced ferroptosis [27]. Early reports have indicated that EMP1 activates Rac1 through the copine-III-mediated intracellular signal [28]. Therefore, the expression of ferroptosis-associated proteins was separately examined via Western blot analysis. It is observable that overexpression of EMP1 remarkably upregulated the expression of Rac1 while it did not affect the levels of GPX4, NOX4, and ACSL4 compared with the control group (Figure 4(a)). Overexpression of EMP1 enhanced the Rac1 expression at mRNA level in Hep2 and Detroit562 cells (Figure 4(b)). Next, we sought to identify how Rac1 regulates RSL3 sensitivity in HNSCC. Furthermore, cells were treated with Rac1 activator PMA and Rac1 inhibitor NSC 23766, respectively, and CCK-8 assay was used to detect the cell viability. We found that PMA could reverse RSL3 sensitivity caused by EMP1 overexpression (Figures 4(c) and 4(d)). The level of oxygen-free radical indicated that PMA could reverse intracellular ROS activity was demonstrated by the membrane-permeable fluorescent probe DCFH-DA (Figures 4(e) and 4(f)). Previous studies have shown that Rac1 activation can trigger NOX1-dependent ROS generation [29]. To test whether Rac1 induced the production of ROS contributes to NOX1, we first measured intracellular NOX1 levels. Consistently, overexpression EMP1 increased the level of NOX1, and PMA treatments significantly enhanced the NOX1 expression at mRNA level (Figures 4(g) and 4(h)). These results indicated that EMP1 regulated the RSL3-induced ferroptosis sensitivity via Rac1.

### 3.4. EMP1 Enhancement Blunts HNSCC Cell Resistance to Gefitinib by Promoting Ferroptosis

**Figure 5:**
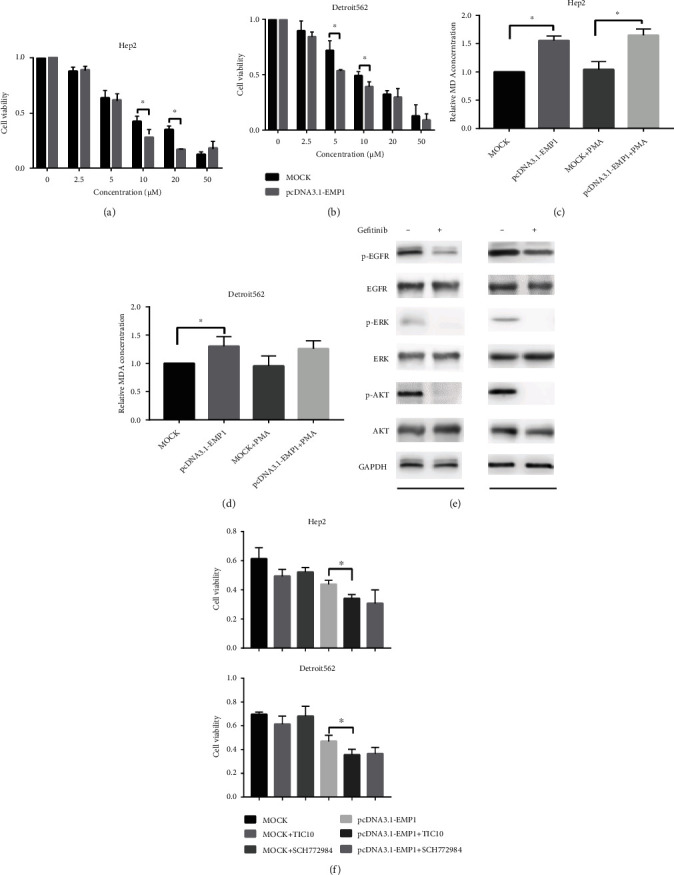
EMP1 enhancement blunts HNSCC cell resistance to gefitinib by promoting ferroptosis. Gefitinib was pretreated in the MOCK- and EMP1-overexpressing Hep2 (a) and Detroit562 (b) cells at different dose for 72 h; then cell viability was detected. MDA levels were detected using a lipid peroxidation MDA assay kit after the treatment of gefitinib (10 *μ*M) in the MOCK- and EMP1-overexpressing Hep2 (c) and Detroit562 (d) cells for 72 h. (e) Western blotting analyzed the effect of gefitinib treatment on including p-EFFR, EGFR, P-ERK, ERK, p-AKT, and AKT protein level in the MOCK- and EMP1-overexpressing Hep2 and Detroit562. (f) Cell viability analyzed the effect of ERK inhibitors TIC10 and SCH772984 in the MOCK- and EMP1-overexpressing Hep2 and Detroit562. Values are the mean ± SD. ^∗^*p* < 0.05.

A previous study reported that EMP1 was a potential biomarker of gefitinib resistance [30]. Clinical trial data show that gefitinib is well tolerated in patients with a wide range of tumor types [31]. We next sought to explore whether EMP1 could influence gefitinib resistance. To illustrate the function of gefitinib in HNSCC, we first used gefitinib to treat cells and the results indicated that the cell death increased mildly with the increasing concentration of gefitinib. Moreover, overexpression EMP1 could improve gefitinib-induced cell death (Figures 5(a) and 5(b)). The MDA level in HNSCC cells after treatment with gefitinib at different concentrations was also determined using an MDA assay kit. As exhibited in Figures 5(c) and 5(d), gefitinib dose-dependently increased MDA concentration. Gefitinib (10 *μ*M) was then selected to treat HNSCC cells for the following experiments, and it induced significant difference between pcDNA3.1-EMP1 and the control groups. Gefitinib is a targeted therapy drug that takes effect by inhibiting the EGFR pathway. The downstream signaling proteins involving several signal transduction cascades, such as MAPK, Akt, ERK1/2, and JNK pathways, are triggered by EGFR autophosphorylation. To verify whether EMP1 enhancement to the gefitinib-induced ferroptosis was directly through the above pathways, protein levels including p-EFFR, EGFR, P-ERK, ERK, p-AKT, and AKT were determined. The result of Figure 5(e) indicated that overexpression EMP1 dramatically reduced the levels of p-EGFR, p-ERK, and p-AKT after gefitinib treatment relative to the control group. Additionally, to study whether ERK phosphorylation plays a key role in gefitinib resistance, we used ERK inhibitors, TIC10 and SCH772984, to interfere with Hep2 and Detroit562 cell lines separately. The results of cell viability assay indicate that both inhibitor treatments did not affect the cell death. However, the combination of overexpression and TIC10 completely facilitates the gefitinib-induced cell death (Figure 5(f)), indicating that the ERK signaling is necessary for gefitinib resistance and ERK inhibition combined with EMP1-abrogated gefitinib resistance. Summing up, these data suggest that gefitinib suppresses cell viability and overexpression EMP1 promotes gefitinib sensibility to ferroptosis in HNSCC cells.

## 4. Discussion

Although HNSCC patients benefit from screening and standard chemotherapy, they still face high incidence and recurrence rates, leaving HNSCC a major challenge in the clinical setting [32, 33].

Ferroptosis induction is widely used to inhibit tumorigenesis [34]. Brent et al. found several small compounds could kill RAS mutant cancer cells, such as erastin and RSL3. These compounds kill cancer cell in iron-dependent manner which is named as ferroptosis. Mechanistically, erastin inhibits cystine/glutamate antiporter (system xc^−^) and RSL3 targets to GPX4 [16]. EMP1 has moved into the limelight in these tumor entities, where its expression is controversial in multiple cancer types; however, its expression and function are contradictory. A previous study has corroborated that EMP1 serves as a promising oncogene and is obviously downregulated in tumors and can enhance cell cytotoxicity in various cancer cells [35]. The underlying mechanisms of EMP1 ability in inducing ferroptosis have not been elucidated. In the present study, our results indicated that EMP1 significantly induced cell death in HNSCC cell lines, and overexpression of EMP1 could not induce ferroptosis directly but facilitated RSL3-induced ferroptosis. Moreover, EMP1 promoted Rac1 expression and was involved in NOX1 signaling pathway. EMP1's promotion of gefitinib sensitivity was ERK signal pathway-dependent. These findings could provide a new theoretical basis for the EMP1-induced destructive effects of cells and contribute to therapeutic drug development for HNSCC in the future.

In this study, we first utilized the public databases to investigate the expression of EMP1 in HNSCC and the results showed that EMP1 was expressed at low levels in cancer. The *in vitro* experiment results are consistent with previous observations. Combining the significantly enriched iron ion binding signal pathway between EMP1-low and high groups, we speculate that maybe there was activated ferroptosis in HNSCC cancer that could induce cell death. To verify this, we investigated the relationship between overexpression of EMP1 and ferroptosis. Even though overexpression of EMP could not induce ferroptosis directly, but further study revealed that overexpression of EMP1 enhanced RSL3-dependent cell death. However, EMP1 did not affect the cellular response to erastin-induced ferroptosis; the underlying mechanism is not clear. Recently, erastin was also found to promote autophagy, and in HPV-positive HNSCC with different levels of autophagy inhibition could induce resistance against ferroptosis [36, 37]. Therefore, there was an activated autophagy pathway in HNSCC cancer that could antagonize erastin-induced ferroptosis [38]. Whether the detailed mechanisms are consistent still needs our further investigation.

Ferroptosis, as a new form of cell death, is characterized by lipid peroxidation. We next explored whether EMP1 could enhance lipid peroxidation induced by RSL3, and the results showed that overexpression of EMP1 significantly increased lipid peroxidation level and reactive oxygen species level in a dose and time-dependent manner, which means that the effects of EMP1 could be related to the cell density related Hippo-YAP/TAZ pathway. Besides, EMP1 mRNA was reported downregulated upon TAZ knockdown [24]. We then explored whether there was a connection between EMP1-regulated ferroptosis and Hippo-YAP/TAZ pathway. Mechanistically, the function of EMP1 is controlled by the upstream Hippo-YAP/TAZ pathway, especially YAP1. EMP1 has been reported to be downregulated in HNSCC and correlated with lymphatic metastasis [39–41]. Overexpression of EMP1 could induce apoptosis and inhibit cell migration and invasion, while restoring EMP1 expression rescued cell growth, clonogenic potential, and invasive trait of cervical carcinoma cell [42]. Furthermore, a series of cell density tests elucidated the contribution of TAZ/EMP1 axis in cancer progression [24]. These findings imply that EMP1 may function as a tumor suppressor in certain cancers.

Of interest, cancer cells' ability in adapting to an oxidative environment to control ferroptosis may reshape the tumor niche that facilitates tumor growth and progression. EMP1 is suggested to regulate cellular oxidative status, and increased cellular ROS has been reported to cause lipid peroxidation [24]. Recent evidence indicates that upregulation of EMP1 could enhance cell migration and invasiveness by activating a small GTPase Rac1 [43, 44]. Chen et al. presented a model that Rac1 activation provided an essential effector for NOX1-dependent ROS generation [45]. The RAC1-NOX1 complex was also reported to be able to induce ROS generation and cellular senescence [46, 47]. Thus, we first investigated the relationship between EMP1 and Rac1, and the results showed that EMP1 was also found to be involved in mediating Rac1 expression. Notably, NOX1 has also been shown to be mediated by Rac1. Herein, these findings reveal a molecular link between EMP1 and Rac1 in the control of ferroptosis in HNSCC and a possible treatment strategy for HNSCC by promoting Rac1.

Importantly, emerging evidence has implicated ferroptosis in chemoresistance in several cancers [48–50]. A large number of recent studies have shown the emergence of gefitinib resistance through a variety of mechanisms. The main elucidation is that cancer cell could promote proliferation by inhibiting the EGRF activity [51–53]. A previous study has shown that durable cancer cells that are resistant to drugs are prone to ferroptosis [54]. Therefore, an understanding of the cellular targets of gefitinib will allow the discovery of biomarkers for predicting outcomes and provide information for overcoming gefitinib resistance in HNSCC. ERK signaling plays a crucial role in the resistance of NSCLC, and ERK activation requires dual phosphorylation of specific MAPK kinase [55]. Chen et al. also found that reducing the activity of the ERK signaling pathway can restore the sensitivity of cells to gefitinib [56]. Another study has proved that EMP1 is a biomarker of gefitinib resistance, and the resistance mechanism is independent of the EGFR somatic mutations [31]. We then explored whether there was a connection between EMP1-regulated ferroptosis and gefitinib resistance in HNSCC. We found that overexpression of EMP1 enhanced gefitinib sensitivity to ferroptosis in HNSCC and this effect was related to the activation of ERK signal pathway. Therefore, we determined the role of ERK-mediated ferroptosis in the emergence of gefitinib resistance in HNSCC cells and suggested that EMP1 could sensitize HNSCC cells to gefitinib by ferroptosis induction via blocking p-ERK activation.

**Figure 6:**
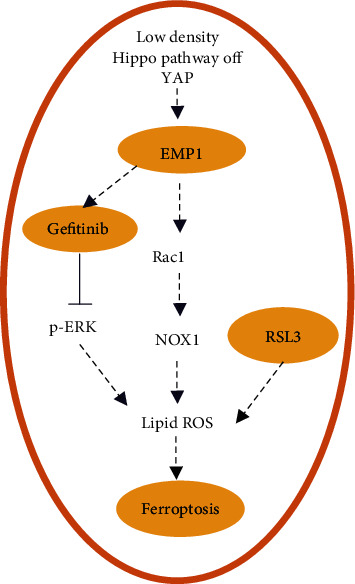
Schematic abstract of the possible underlying mechanism of EMP1 promotion RSL3-induced ferroptosis sensitivity by controlling the Rac1/NOX1 in HNSCC cells. EMP1 overexpression enhanced the resistance to gefitinib by promoting ferroptosis via ERK pathway.

In conclusion, this study confirmed the role of EMP1 in RSL3-induced ferroptosis and gefitinib resistance in HNSCC and preliminarily explored the underlying mechanism during the regulation process. Thus, the present research may implicate EMP1 as a promising therapeutic candidate to overcome chemoresistance in HNSCC Figure 6.

## Data Availability

The data that support the findings of this study are available from the corresponding author upon reasonable request.
